# Gene Expression Analysis of Mouse Embryonic Stem Cells Following Levitation in an Ultrasound Standing Wave Trap

**DOI:** 10.1016/j.ultrasmedbio.2010.10.019

**Published:** 2011-02

**Authors:** Despina Bazou, Roisin Kearney, Fiona Mansergh, Celine Bourdon, Jane Farrar, Michael Wride

**Affiliations:** ∗ Centre for Research on Adaptive Nanostructures and Nanodevices (CRANN), Trinity College Dublin, Dublin, Ireland; † Department of Zoology, Trinity College Dublin, Dublin, Ireland; ‡ Smurfit Institute of Genetics, Trinity College Dublin, Dublin, Ireland

**Keywords:** Embryonic stem cells, Embryoid bodies, Gene expression, Differentiation, Neural, Pluripotency, Ultrasound, Cell manipulation, Microenvironment

## Abstract

In the present paper, gene expression analysis of mouse embryonic stem (ES) cells levitated in a novel ultrasound standing wave trap (USWT) (Bazou et al. 2005a) at variable acoustic pressures (0.08–0.85 MPa) and times (5–60 min) was performed. Our results showed that levitation of ES cells at the highest employed acoustic pressure for 60 min does not modify gene expression and cells maintain their pluripotency. Embryoid bodies (EBs) also expressed the early and late neural differentiation markers, which were also unaffected by the acoustic field. Our results suggest that the ultrasound trap microenvironment is minimally invasive as the biologic consequences of ES cell replication and EB differentiation proceed without significantly affecting gene expression. The technique holds great promise in safe cell manipulation techniques for a variety of applications including tissue engineering and regenerative medicine. (E-mail: Bazoud@tcd.ie)

## Introduction and Literature

Cell manipulation techniques are important in many areas of research including cell biology, molecular genetics, biotechnological production, clinical diagnostics and therapeutics. Physical methods of manipulating suspended cells at single-particle microscopic resolution include hydrodynamic (Lin et al. 2008), optical (Mohanty et al. 2008; Bustamante et al. 2009), dielectrophoretic (Jang et al. 2009; Thomas et al. 2009), magnetic (Koschwanez et al. 2007; Liu et al. 2009) and ultrasonic (Bazou et al. 2005a; Evander et al. 2007; Oberti et al. 2007) cell trapping.

Of the above mentioned methods, ultrasound trapping has been less extensively exploited. Compared with other methods, ultrasonic cell manipulation is an inexpensive noncontact technique that allows simultaneous and synchronous manipulation of a large number of cells in a very short time (Bazou et al. 2005a). It is simple in both set-up and operation and is noninvasive, chemically inert (nontoxic) and physically nondestructive (Kim et al. 2004). Taking into account its high efficiency and reliability and the fact that it can be used with the majority of cell types, this technique holds great promise in cell manipulation techniques for a variety of applications.

We have previously reported (Bazou et al. 2005a) on a novel two-dimensional (2-D) ultrasound standing wave trap (USWT) capable of holding >10,000 cells at the focal plane of a microscope. The USWT is an ultrasound resonator where the acoustic path-length in the cell suspension is a single half wavelength. The resonator has a pressure node plane half way through the cell suspension and parallel to the transducer (Bazou et al. 2005a, 2005b). The cell trap exploits the fact that cells experience an axial direct acoustic radiation force when in an ultrasound standing wave field (Bazou et al. 2005a, 2005b). This force drives them toward a node plane. They then move, within that plane, to accumulate at the centre of the field, *i.e*., at the nodal plane (Coakley et al. 2003). The USWT has been used to synchronously and rapidly (within 10 s of seconds) form and levitate 2-D (Coakley et al. 2003; Bazou et al. 2005a, 2005b) and three-dimensional (3-D) (Liu et al. 2007; Bazou et al. 2008) cell aggregates in suspension away from the influence of solid substrata. The technique has provided data on the intracellular temporal progression of F-actin formation (Bazou et al. 2005a) as well as on the gap junctional intercellular communication (Bazou et al. 2006) in a large (ca. 10^4^) sample of cells.

A frequently discussed matter in ultrasound trapping is the viability of trapped cells after being exposed to ultrasound. Nyborg (2001) reviewed the 80-year history of studies of biologic effects of ultrasound that had been conducted as there is great interest in applications of ultrasound to biotechnology and medical therapy. The need to assess the safety of the widespread medical applications of ultrasound was also highlighted (Nyborg 2001). He investigated the thermal effects that can arise because of sound absorption, effects due to cavitation as well as phenomena that arise due to acoustic radiation force or torque or acoustic streaming. In line with Nyborg’s review (2001), we have previously examined the physical environment of the USWT (Bazou et al. 2005a). The results of the latter study, as well as those reported by Bazou et al. (2005b, 2006, 2008) and Edwards et al. (2007) showed that the ultrasound trap does not compromise cell behaviour or cell viability (cells remained 99% viable over 1 h of continuous levitation in the ultrasound trap), therefore, the standing wave operates only to concentrate cells locally as in tissue. However, data with regard to the effects of ultrasonic cell manipulation on gene expression profiles of cells has been limited to date.

In this study, we investigate for the first time the influence of ultrasonic cell manipulation on key genes expressed during differentiation of embryonic stem (ES) cells (Table 1). ES cell differentiation *in vitro* is a model for early embryonic development (Mansergh et al. 2009). During this developmental period, embryonic gene expression patterns may be liable to aberrant programming (Lonergan et al. 2006). Embryos can exhibit plasticity in their ability to adapt to suboptimal *in vitro* conditions (Lonergan et al. 2006); however, their sensitivity to their environment can lead to long-term alterations in the characteristics of foetal and postnatal growth and development; it is thus important to investigate the effect (if any) of ultrasound in the context of early ES cell pluripotency and differentiation.

## Materials and Methods

### Cell culture

The IMT11 embryonic stem (ES) cell line, derived from 129Sv mice was used for all experiments described in this study. This cell line was a kind gift of Professor Sir Martin Evans (Cardiff University). This cell line was selected as it is not genetically modified and its gene expression profile has already been studied *via* microarray during expansion and early differentiation (Mansergh et al. 2009). Undifferentiated ES cells were maintained at 37°C in a humidified atmosphere with 5% CO_2_ on 0.1% gelatin in DMEM, with 2 mM L-glutamine, 50 U/mL penicillin, 50 μg/mL streptomycin (all from Gibco; Invitrogen Ltd, Paisley, Renfrewshire, UK), 10^−4^ β-mercaproethanol (Merck kGaA; 64293 Darmstadt, Germany), 10^−3^ U/mL murine LIF (ESGRO TM; Invitrogen Ltd, Paisley, Renfrewshire, UK), 10% foetal calf serum (FCS) and 10% newborn bovine serum (NBS). For the generation of embryoid bodies (EBs) a semiconfluent 100 mm dish of ES cells was trypsinized (0.25% trypsin/EDTA, Invitrogen), followed by trituration in additional ES medium to achieve a single cell suspension. ES medium was prepared as above for + LIF EBs and without LIF for –LIF differentiations.

### Ultrasound trap

The in-house constructed trap employed in the present work had four layers; a transducer (Ferroperm, Kvistgard, Denmark) nominally resonant in the thickness mode at 3 MHz and mounted in a radially symmetric housing, a steel layer coupling the ultrasound to a one half wavelength (λ/2 or 0.25 mm depth, where λ is the wavelength of sound in water at 3 MHz) aqueous layer and a quartz acoustic reflector that provided optical access from above (Bazou et al. 2005a). The outer diameter of the cylindrical steel body was 35 mm. The “sample-containing” active area had a diameter of 18 mm. The disc transducer (12 mm diameter) was driven at 2.13 MHz. Its back electrode was etched to a 6 mm diameter circle so as to give a single central aggregate in a single half-wavelength chamber. The quartz glass acoustic reflector had a thickness of 0.5 mm (λ/4) so as to locate the single pressure node plane half way through the sample volume. The piezoceramic transducer was driven from a function generator (Hewlett Packard 33120A; Hewlett Packard, Berkshire, UK) to generate a mechanical wave.

### Optical system

A fast, high-resolution XM10 (Soft Imaging System, SIS, GmbH, Munster, Germany) mounted on an Olympus BX51M reflection epi-fluorescence microscope allowed observation in the direction of sound propagation (negative z-axis) (Bazou et al. 2005a). Images were captured by a standard PC equipped with the Cell-D image acquisition and processing software (Soft Imaging System, SIS, GmbH).

### Experimental procedure

Single cell suspensions of ES cells were prepared as described above and diluted to 3000 cells/μL. The ultrasound trap was placed into the tissue culture cabinet to ensure sterility of the samples. A stereo-microscope (Swift Instruments International, San Jose, CA, USA), on which the ultrasound trap was placed, was also inserted into the tissue culture cabinet to monitor the aggregate growth process. Cell suspensions were introduced into the trap (pre-coated with gelatin to inhibit any cell-substratum interactions) at room temperature with a sterile 2 mL syringe (Plastipak, Becton Dickinson, Oxford, UK). The acoustic field was initiated and aggregates were allowed to form. Two sets of samples were generated.

The first set of samples was levitated in the trap at 0.08 MPa (the minimal pressure at which aggregates remained levitated in suspension) and 0.85 MPa (the maximum pressure achieved with the current experimental set-up) for 5 min to determine whether the acoustic pressure affects gene expression. The trap was driven at its resonance frequency of 2.14 MHz. Experimental treatments included: (1) control (C) (cells not introduced into the trap-untreated), (2) control trap (CT) (cells were introduced into the trap but the ultrasonic field was off), (3) low acoustic pressure (L) (cells were levitated in the trap at 0.08 MPa) and (4) high acoustic pressure (H) (cells were levitated in the trap at 0.85 MPa).

In the second set of samples, the acoustic pressure was kept constant at 0.85 MPa, while the time of levitation varied between 5 and 60 min, to examine whether long periods of levitation in the trap at the highest acoustic pressure affects gene expression. The trap was again driven at its resonance frequency of 2.14 MHz. Experimental treatments were as follows: (1) control (C) (cells were not introduced into the trap-untreated), (2) control trap 5 min (CT5) (cells were introduced into the trap while the ultrasonic field was off for 5 min), (3) control trap 60 min (CT60) (cells were introduced into the trap while the ultrasound field was off for 60 min), (4) ultrasound 5 min (US5) (cells were levitated in the trap at 0.85 MPa for 5 min) and (5) ultrasound 60 min (US60) (cells were levitated in the trap at 0.85 MPa for 60 min).

The ultrasound field was subsequently switched off and aggregates were slowly recovered from the trap with a syringe. They were then dispersed back into single cell suspensions (as excessive aggregation results in spontaneous differentiation) and maintained, as appropriate for ES and EBs, in culture until they reached 70% to 80% confluence prior to further analysis. Specifically, one batch of the ultrasound levitated ES cells was plated in gelatin-coated Petri dishes for proliferation, whereas the second batch of ES cells was plated in nonadherent bacterial Petri dishes without LIF for differentiation. This involves nonadherent ES cells aggregating randomly and forming EBs of different sizes spontaneously in culture. EBs were fed every 2 days and cultured for 4 days (D4 EBs) in the absence of LIF. Retinoic acid (RA), as specified by Bibel et al. (2007) was then added to induce early neural differentiation (Bain et al. 1995; Bibel et al. 2007) and cells were maintained in culture for an additional 4 days (D8 EBs). All experiments were repeated three times (three replicates/set of experiment, *i.e*., a total of nine samples were overall assessed) and representative data are presented, unless otherwise stated.

### Karyotype analysis

Karyotype analysis was performed on the ES cell samples: C, CT, L and H to examine whether the acoustic pressure affects chromosomal stability. Analysis was performed as previously described (Mansergh et al. 2005). Scoring of cells with chromosome numbers varying between <39 and >41 was then performed through microscopic observations. The number of cells with 40 chromosomes was divided to the total number of cells in at least five randomly selected fields of view.

### Total RNA extraction and reverse transcription

Cells were rinsed with ice cold phosphate buffered saline (PBS) and resuspended in 1 mL of Tri reagent (Sigma Aldrich, Poole, UK). The TRI Reagent was used according to the manufacturer’s protocol (Sigma Aldrich) for RNA extraction, followed by OD 260/280 spectrophotometry (NanoDrop ND-1000, Thermo Scientific, Wilmington, DE). Samples were DNase treated using the DNA-free kit (Applied Biosystems, Warrington, UK) as per manufacturer’s instructions and subsequently reversed transcribed using the random hexamer protocol of the Superscript First Strand Synthesis System for RT-PCR (Invitrogen). RT reactions were diluted with nuclease free water (Ambion) to 100 μL before polymerase chain reaction (PCR) analysis. A “no RT” control corresponding to each sample was also produced for all RT-PCR experiments; these were treated in exactly the same way as the samples except that reverse transcriptase was not added.

### qPCR

qPCR was carried out according to the QuantiTect SYBR Green protocol (Qiagen, Crawley, UK), using an ABI 7500 cycler (Applied Biosystems). The following samples were tested: ES cells (C, CT5, CT60, US5, US60) and EBs (C, CT, L, H at days 4 and 8). qPCRs were carried out in 20 μL volumes using 10 μL of 2× QuantiTect SYBR green PCR master mix, 10 pmol/μL of each primer set and 25 ng cDNA per reaction. Primers used were as listed in Table 2.

### Western blot

Western blot analysis was performed in the ES cell samples C, CT5, CT60, US5 and US60. Samples were rinsed with ice cold PBS and suspended in 1× RIPA buffer. Protein concentration was determined using the Bradford assay (Biorad Laboratories, Hertfoshire, UK). The samples were boiled in the SDS sample buffer for 5 min and were subjected to SDS-PAGE, followed by Western blotting with the primary antibodies goat monoclonal anti-mouse Nanog (1:2000; R and D Systems, Abingdon, UK), goat polyclonal Oct4 (1:1000; AbCAM, Cambridge, UK), while the secondary antibody was rabbit polyclonal to goat IgG-horseradish peroxidise-conjugated (1:20,000; AbCAM).

### Immunofluorescence

Immunofluorescence was performed on the ES cell samples C, CT5, CT60, US5 and US60. Samples were for this purpose grown on 35 mm in a 24-well plate. Samples were fixed with 4% paraformaldehyde for 15 min, rinsed with saline and subsequently serum-blocked (Sigma) for 30 min. The primary antibodies (Oct4 and Nanog [both at 10 μg/mL]) were added for 1 h at room temperature in the dark, followed by the addition of donkey anti-goat Cy3 (5 μg/mL; Invitrogen Ltd., Paisley, Refrewshire, UK) for 1 h at 4°C. Samples were further rinsed with saline and mounted in Vectashield (Vector, Peterborough, UK) prior to microscopic examination.

### Statistical analysis

The data presented here are shown as mean ± standard error of mean. Each experiment was repeated at least three times (three replicates/set of experiment, *i.e*., a total of nine samples were overall assessed). Representative data are presented. Analysis of means was performed with a one way analysis of variance (ANOVA) (GraphPad Prism). Differences were considered significant at *p* values less than 0.05.

## Results

### Effect of acoustic pressure on ES cell and EB gene expression

#### qPCR

**Fig. 1:**
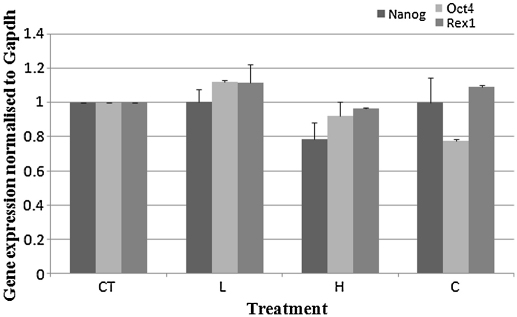
qPCR analysis of embryonic stem (ES) cell pluripotency genes normalised to the Gapdh housekeeping gene expression. Treatments have been normalised with respect to the CT values. Error bands indicate one standard error of the mean. Mean was determined from three repetitions in each case.

Initially, the effect of varying the acoustic pressure in the ultrasound trap on the genetic profile of ES cells and EBs was examined. Cells were levitated in the ultrasound trap for 5 min at 0.08 (L) and 0.85 MPa (H). Their gene expression profile was assessed using qPCR (Fig. 1). All data presented here have been normalised with respect to the CT treatment to rule out an effect of the ultrasound trap itself on gene expression, as cells subjected to CT, L and H treatments have all undergone the same preparation and introduction into the ultrasound trap processes.

#### ES cell gene expression

No significant difference in the expression of the three pluripotency genes (*p* > 0.05) was observed for ES cells levitated in the ultrasound trap for 5 min at 0.08 (L) and 0.85 MPa (H), respectively (Fig. 1).

#### EB gene expression

**Fig. 2:**
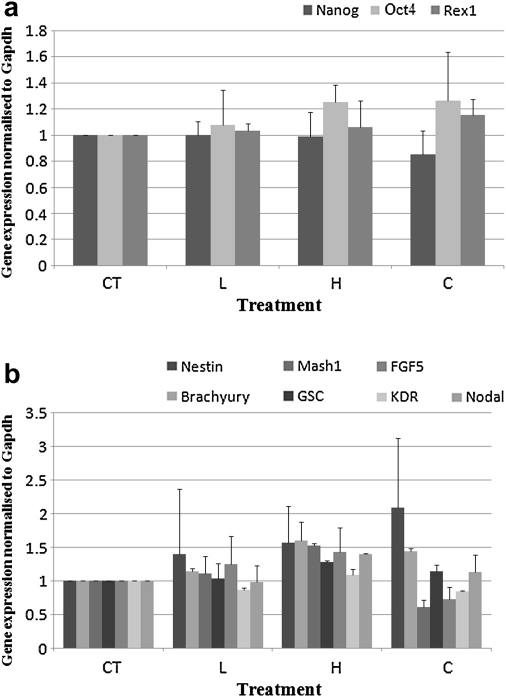
qPCR analysis of the (a) pluripotency and (b) early differentiation genes normalised to the Gapdh housekeeping gene expression in D4 embryoid bodies (EBs). Treatments have been normalised with respect to the CT values. Error bands indicate one standard error of the mean, determined from three repetitions in each case.

**Fig. 3:**
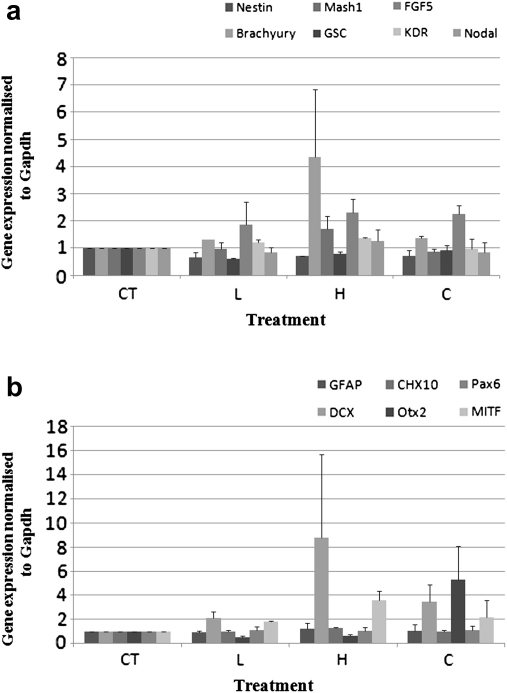
qPCR analysis of the (a) early and (b) late differentiation genes normalised to the Gapdh housekeeping gene expression in D8 embryoid bodies (EBs). Treatments have been normalised with respect to the CT values. Error bands indicate one standard error of the mean, determined from three repetitions in each case.

With the exception of the early differentiation gene *Mash1* (*p* 0.0409 < 0.05), there was no significant difference in the expression of the pluripotency and early differentiation genes in D4 EBs (Fig. 2a and b). Similarly, in D8 EBs no significant difference could be detected in the expression of all genes investigated under the four treatments (Fig. 3a and b). These data also confirmed our semiquantitative PCR pilot analysis (data not shown).

### Karyotype analysis

No difference could be detected in the chromosome number of ES cells subjected to the aforementioned treatments (C, CT, L, H) as assessed by microscopic observation and subsequent counting (data not shown).

### Effect of duration of ultrasonic levitation on ES cell gene expression

#### Semiquantitative PCR

**Fig. 4:**
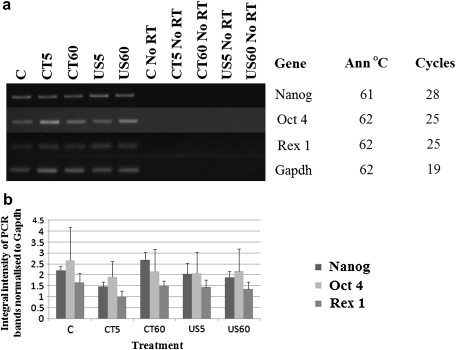
(a) Semiquantitative PCR analysis of the pluripotency genes normalised to the Gapdh housekeeping gene expression in embryonic stem (ES) cells levitated in the trap for 5 and 60 min at 0.85 MPa. The “no RT” samples are also shown together with the PCR conditions. (b) Integral intensity measurements of the PCR bands shown in (a) normalised to the Gapdh housekeeping gene expression.

As the data obtained from our qPCR studies (and from our semiquantitative PCR pilot study, data not shown) revealed no significant effect (with the exception of *Mash1*) of the acoustic pressure on the gene expression profile of ES cells as well as EBs, we proceeded in asking the question as to whether levitating cells at the highest employed acoustic pressure (0.85 MPa) for a maximum of 60 min modifies gene expression. In this series of experiments, ES cells were used due to their ease of culture and less time required for cell culture in comparison with EBs. Samples were as follows: C, CT5, CT60, US5 and US60. Our results (Fig. 4a) showed that there is no significant difference in the integral intensity of the PCR bands (normalised to Gapdh) of the different treatments (Fig. 4b). The *p* values were: Nanog (*p* = 0.17), Oct4 (*p* = 0.99) and Rex1 (*p* = 0.71).

#### qPCR

**Fig. 5:**
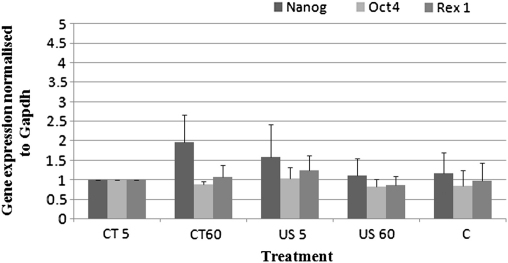
qPCR analysis of the pluripotency genes normalised to the Gapdh housekeeping gene expression in ES cells levitated in the trap for 5 and 60 min at 0.85 MPa. Treatments have been normalised with respect to the CT values. Error bands indicate one standard error of the mean.

Confirmation of the above results was obtained through qPCR (Fig. 5). No significant difference in the expression of the three pluripotency genes investigated could be detected between the different treatments (*p* > 0.05 for all three genes).

### Western blot analysis of ES cells

**Fig. 6:**
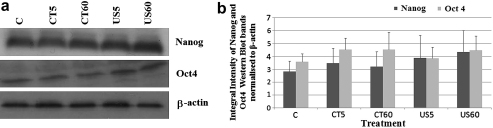
(a) Western blot analysis of Nanog and Oct4; both proteins where highly expressed in all treatments. β-actin was used for normalization. Data are representative of three independent experiments. (b) Integral intensity measurements of the western blot bands shown in (a) normalised to β-actin. No significant differences could be detected in the expression of both proteins by embryonic stem (ES) cells subjected to the various treatments.

Western blotting was used to investigate protein expression of ES cells levitated for a maximum of 60 min in the trap at 0.85 MPa. Bands were detected at the molecular weight indicative of the two pluripotency proteins: 45 KDa for Oct4 and 34 KDa for Nanog (Fig. 6a). A similar banding pattern throughout the different treatments (C, CT5, CT60, US5 and US60) was observed (Fig. 6a) in the blot. Integral intensity measurements of the Western blot bands normalised to those obtained from the β-actin revealed no significant difference in protein expression between the different treatments (*p* > 0.05 for both genes) (Fig. 6b).

### Immunofluorescence analysis of ES cells

**Fig. 7:**
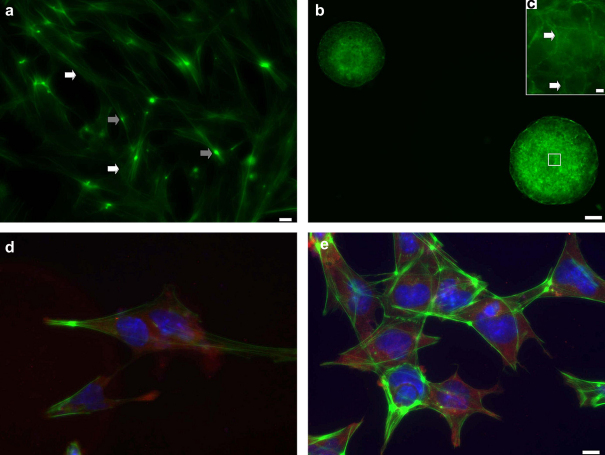
Representative micrographs captured from different fields of view of the distribution of (a, b, c) F-actin, (d) Nanog and (e) Oct4 in embryonic stem (ES) cells levitated in the trap for 5 and 60 min at 0.85 MP. (a) Striking stress fibres (white arrows) and focal spots (grey arrows) were observed in single ES cells. Scale bar is 5μm. (b) Some ES cells formed embryoid bodies (EBs) with extensive cell-cell contacts seen through staining of the F-actin cytoskeleton. Scale bar is 50 μm. (c) Close-up of the F-actin staining in EBs shown in (b) accumulated at sites of cell-cell contact (arrows). Scale bar is 5 μm. (d) and (e) Triple-staining images showing the cytoplasmic distribution of Oct4 (d) and (e) Nanog (AlexaFluor Cy3-red dye), Filamentous (F-) actin (Phalloidin 488-green dye) and nucleus (DAPI-blue dye). Scale bar is 10 μm.

Following levitation in the ultrasound trap at 0.85 MPa for 5 and 60 min, ES cells were plated and allowed to grow until they reached confluence as described in materials and methods. Microscopic observations showed that during culture ES cells spread in a fibroblastic manner as revealed by immunostaining of the F-actin cytoskeleton. Striking stress fibres (Fig. 7a; white arrows) and focal spots (Fig. 7a; grey arrows) were observed. However, some ES cells formed EBs with extensive cell-cell contacts seen through staining of the F-actin cytoskeleton (Fig. 7b). Figure 7c shows a close-up of the F-actin accumulated at sites of cell-cell contact (Fig. 7c, arrows). Positive expression of Oct4 and Nanog was observed in the immunofluorescent analysis of all samples (CT5, CT60, US5 and US60). Specifically, cytoplasmic distribution of Nanog (Fig. 7d) and Oct4 (Fig. 7e) was detected in ES cells. This staining pattern was detected in control (C) samples and remained as such over the following treatments (CT5, CT60, US5 and US60). No detectable difference could be observed by microscopy in the Nanog and Oct4 distribution pattern within the cells between the various treatments.

## Discussion and Summary

There is strong evidence that the behaviour of stem cells is strongly affected by their local environment or niche (Watt and Driskell 2010). Some aspects of the stem cell environment that are known to influence self-renewal and stem cell fate are: adhesion to extracellular matrix proteins, direct contact with neighbouring cells, exposure to secreted factors and physical factors (such as oxygen concentration and shear stress) (Watt and Hogan 2000; Morrison and Spradling 2008).

The environment to which the mammalian embryo is exposed during the pre-implantation period of development has a profound effect on the physiology and viability of the conceptus (Gardner and Lane 2005). It has been demonstrated that conditions that alter gene expression can also adversely affect cell physiology. It is therefore important to examine the factors contributing to abnormal gene expression and altered imprinting patterns, and whether problems can be arrested by using more physiologic culture conditions (Gardner and Lane 2005). It is also of note that the sensitivity of the embryo to its surroundings decreases as development proceeds. Post compaction and environmental conditions have a lesser effect on gene function as development proceeds. Therefore, we undertook the present study to examine whether the employed ultrasound trap microenvironment does affect stem cell expansion and differentiation, and thus whether ultrasound cell manipulation affects the gene expression profile of stem cells.

### Effect of acoustic pressure on ES cell and EB gene expression

Our results (Figs. 1–3) show that, with the exception of *Mash1*, the gene expression profile of ES cells and EBs was not influenced following levitation of cells at the highest employed acoustic pressure (0.85 MPa). Furthermore, no effect on stem cell karyotype was observed (data not shown).

We have previously calculated that the attractive acoustic force between ultrasonically agglomerated cells of 10 μm diameter equals the van der Waals force at surface separations of 34 nm (Coakley et al. 2003) when the pressure amplitude is 0.25 MPa in a 1.5 MHz trap. For the 14 μm diameter, ES cells examined in the present study the acoustic force at a pressure amplitude of 0.85 MPa (H), equals the van der Waals force at a surface separation of 43 nm. This distance is greater than the range of surface receptor molecules. At smaller separations, the van der Waals force dominates the essentially constant acoustic force. When the pressure amplitude is reduced to 0.08 MPa (L) during aggregate levitation, the acoustic interaction is less than the van der Waals force at separations less than 237 nm and is negligible at the surface separation at which receptors operate. Therefore, any gene expression change would be most likely attributed to cell-cell interactions rather than to any “stress” imposed on cells levitated in the trap at the highest acoustic pressure.

However, *Mash1* was the only gene out of the 16 genes examined, found to be differentially regulated (though statistically marginally different *p* 0.0409 < 0.05), in D4 EBs (Fig. 2b) but not in D8 EBs (Fig. 3a). More specifically, the expression of this gene was upregulated with increasing acoustic pressure, while in the CT and C treatments its expression was at its lowest. As levitation of ES cells at the highest acoustic pressure for 60 min had no effect on the expression of the pluripotency genes (Figs. 4 and 5), we suggest that inherent differences between ES cells and EBs might account for the upregulation of the *Mash 1* gene instead of any direct effect of the acoustic field. ES cells are cultured in a planar format (monolayer, 2-D architecture) and are thus provided with a more defined substrate for their attachment and uniform exposure to soluble media components (Bratt-Leal et al. 2009). EBs on the other hand, are of a 3-D architecture whereas their size, shape and homogeneity varies even between EBs of the same culture while they are highly sensitive to soluble media components (Mansergh et al. 2009); consequently, their culture conditions and environment are not as defined as those of ES cells. Furthermore, as reported by Mansergh et al. (2009), there is large variability between batches of EBs and indeed between individual EBs themselves, thus reasoning the statistically marginal upregulation of *Mash1* expression in D4 EBs.

### Effect of duration of ultrasonic levitation on ES cell gene expression

The gene expression profile of ES cells levitated in the ultrasound trap at an acoustic pressure of 0.85 MPa for 5 and 60 min is shown in Figures 4 and 5. No significant difference in the expression of the three pluripotency genes Nanog, Oct4 and Rex1 was observed between the treatments.

We decided to set 60 min as the maximum time period of levitation as: (1) this time period has been the maximum one employed by us previously (Bazou et al. 2005b, 2006) and (2) cell-cell interactions have been shown to have reached their equilibrium state through expression of cell membrane surface receptors (Bazou et al. 2006).

Our Western blotting data (Fig. 6) showed that the amount of protein expressed by ES cells is not affected by 60 min levitation in the trap at 0.85 MPa (*p* = 0.936 and 0.931 for Nanog and Oct4, respectively). We note that we selected two (Nanog and Oct4) of the three pluripotency genes as a good indication of their expression at the protein level. In concurrence, immunofluorescence analysis revealed high expression of Nanog and Oct4 at all experimental conditions (Fig. 7), indicating that the undifferentiated status of ES cells was preserved and remained unaffected by the ultrasound trap microenvironment. Nanog and Oct4 proteins were found present in almost (99%) all single cells as well as in the EBs (data not shown). The pluripotency of ES cells is maintained through continuous high expression of Oct4 and Nanog *in vitro* (Loh et al. 2006; Niwa 2010; Arzumanyan et al. 2009).

In conclusion, the results presented in this study suggest that ultrasonic cell manipulation is a minimally invasive technique where gene expression of mouse ES cells remains unaffected. ES cells within the ultrasound trap microenvironment maintain their pluripotency, while EBs expressed a range of early and late neural differentiation markers. We acknowledge that in the present study a particular cohort of genes was investigated, thus, a cDNA microarray analysis would be the next sensible step. The ultrasound trap acts in a passive manner to concentrate cells locally, while the biologic consequences of ES cell replication and EB differentiation proceeded without affecting expression of the genes examined. As operational conditions are similar to those employed during medical ultrasonography, this study provides further evidence toward the biosafety of ultrasound.

## Figures and Tables

**Table 1:** List of ES pluripotency, early and late differentiation genes

Gene	Identity	Role
*Nanog*	Homeobox transcription factor	Pluripotency
*Oct4*	Homeobox transcription factor	Pluripotency
*Rex1*	Transcription factor	Pluripotency
*Nestin*	Class 6 intermediate filament protein	Neuroectodermal differentiation
*Brachyury*	Transcription factor	Mesodermal differentiation
*Mash1*	Basic helix-loop-helix transcription factor	Neuronal differentiation
*Gsc*	Paired homeobox transcription factor	Spemann organiser and gastrulation movements
*Fgf5*	Fibroblast growth factor	Primitive ectoderm
*Kdr*	Type III receptor tyrosine kinase	Multipotent haematopoietic stem cells
*Nodal*	Member of the TGF-beta superfamily	Anterior-posterior and visceral endodermal patterning
*Gfap*	Component of intermediate filaments of glial cells of the astrocyte lineage	Astrocyte marker
*Dcx*	Microtubule binding protein	Neurogenesis marker
*Otx2*	Bicoid family of homeodomain-containing transcription factors	Vertebrate eye development
*Pax6*	Transcription factor containing both paired box and homeobox binding domains	Central nervous system (CNS) development
*Mitf*	Transcription factor of both the basic helix-loop-helix and leucine zipper family	Early eye development
*Nrl*	Basic motif-leucine zipper transcription factor of the Maf subfamily	Expressed in all cells of the neural retina

ES = embryonic stem.

**Table 2:** Primer sequences for mouse ES pluripotency, early and late differentiation genes

Gene	Oligo	Sequence	Product size (bp)
*Nanog*	Forward	aaaccaaaggatgaagtgcaa	141
	Reverse	gatgcgttcaccagatagcc	
*Oct4*	Forward	atcactcacatcgccaatca	139
	Reverse	ggaaaggtgtccctgtagcc	
*Rex1*	Forward	ctgggtacgagtggcagttt	117
	Reverse	acgtgtcccagctcttagtcc	
*Nestin*	Forward	ccgcttccgctgggtcactgt	227
	Reverse	ctgagcagctggttctgctcct	
*Brachyury*	Forward	catgtactctttcttgctgg	162
	Reverse	ggtctcgggaaagcagtggc	
*Mash1*	Forward	ccacggtctttgcttctgttt	266
	Reverse	tggggatggcagttgtaaga	
*Gsc*	Forward	cagatgctgccctacatgaac	157
	Reverse	tctgggtacttcgtctcctgg	
*Fgf5*	Forward	tgtgtctcaggggattgtagg	136
	Reverse	agctgttttcttggaatctctcc	
Kdr	Forward	tttggcaaatacaacccttcaga	112
	Reverse	gcagaagatactgtcaccacc	
Nodal	Forward	ttcaagcctgttgggctctac	312
	Reverse	tccggtcacgtccacatctt	
*Gfap*	Forward	aaaaccgcatcaccattcct	172
	Reverse	acgtccttgtgctcctgctt	
*Dcx*	Forward	ggccaagagtttctgccaag	244
	Reverse	taatgcagggatcagggaca	
*Otx2*	Forward	aaggagccatgttggactgaa	184
	Reverse	gcctgggaatacaggagcag	
*Pax6*	Forward	ggtccatcaaccagcaacct	212
	Reverse	acaccggatcacctctgctt	
*Mitf*	Forward	gagaaatggcggttagaagca	241
	Reverse	caaccacatgagcaacacaga	
Nrl	Forward	gatggacgatgccctctcac	258
	Reverse	ctgggctactgataaagcacgaa	
*Gapdh*	Forward	caggttgtctcctgcgactt	127
	Reverse	tgctgtagccgtattcattgtc	
*β-actin*	Forward	ccaccatgtacccaggcatt	141
	Reverse	acagtgaggccaggatggag	

ES = embryonic stem.
